# Energy medicine

**DOI:** 10.4103/0973-6131.66770

**Published:** 2010

**Authors:** TM Srinivasan

**Affiliations:** Swami Vivekananda Yoga Anusandhana Samsthana (A Yoga University), No.9, Appajappa Agrahara Chamarajpet, Bangalore - 560 018, India. E-mail: editor@ijoy.org.in

Energy Medicine is a word coined by three researchers who gathered at Boulder, Colorado, USA in the late 1980s. This is defined as any energetic or informational interaction with a biological system to bring back homeostasis in the organism. Meanwhile, in the late 1990s, the National Institutes of Health, the official arm of health policy and implementation in the United States of America defined areas within Complementary and Alternative Medicine through five subdivisions. They are: 1. Mind–Body Medicine, 2. Biologically based practices, 3. Energy Medicine, 4. Manipulative and Body-based practices, and 5. Whole Medical Systems. While these divisions are not arbitrary, it is still breaking up a holistic area into disparate entities. At the core of all this is the concept of subtle energy, which seems to sustain and promote life processes in the biological system.

Thus, subtle energy is another term used along with Energy Medicine. Here the energies activating a person are subtle or of very low intensity. Such low levels may not be measurable at this time. This statement implies that the energies we are talking about are of a physical kind. There are four basic types of energies enumerated in Physics; they are strong and weak forces at the nuclear level, gravitational, and electromagnetic forces. Of these, electromagnetic (or, its equivalent, acoustic) is the only one that is easily manipulated at the present time. Acoustic energy could be transformed into electromagnetic or vice versa through a material property known as piezoelectricity. Many tissues of the body are known to be piezoelectric; hence, any electromagnetic input to the body is transformed into acoustic and any acoustic input could be transformed into electromagnetic energy. Thus, the body is bathed in both electromagnetic and acoustic energies of various frequencies and intensities.[1,2]

There is yet another notion of subtle energy; that is, the energy may not be a physical one. This statement raises many questions: if not physical, what is it? Can it be measured? Is there a physical manifestation of the non-physical energy? Take for example, prayer or non-contact therapeutic touch. In these examples, there is no measurable physical energy that seems to be taking part in the interactions. Although the effects of prayer and therapeutic touch are well-investigated and reported, the energy type and hence the mechanism of action can only be surmised. The energy of chi (or, qi) or prana is not measurable; however, the interaction of chi or prana with the biological system may be deduced. For example, with the appropriate flow of prana, the tissues are healthy; thus, chi gong or yoga may be thought of as stabilizing the flow of prana in the body.

The question arises if subtle energies could at all be measured. Direct measurement of subtle energies is not possible at present as the physical / psychological / spiritual aspects of these energies are not clearly understood in modern terminology. However, indirect measurement of subtle energy in the body is possible through certain physiological correlates that are emerging. Instruments to measure acupuncture activity and electrical discharge photography popularly known as Kirlian photography are the two main contenders for subtle energy monitoring. Acupuncture instruments are based on the observation that acupuncture points have special electrical characteristics; the points have lower resistance to electrical current flow as compared to the surrounding tissues. As each meridian is associated with one or more organs inside the body, the electrical activity of the acupoint seems to be related to the organ function.

In the second kind of instrument, based on Kirlian photography, a high voltage, low current is applied to the finger pads. The colorful discharge that is observed is analyzed in a computer and is related to organ function. Sophisticated instruments are presently available based on these principles and we shall discuss these in more detail in the forthcoming issues. Needless to say, the instruments are undergoing many trials and clinical evaluation, so that their use is acceptable in medical diagnostics and therapy.

Several interesting articles are presented in this issue. It is a privilege for me to be the Editor of IJOY and with all your cooperation, I am sure we can bring the best of scientific investigations in Yoga to many readers around the world.
